# The impact of a 6-week resistance training program with pre- and post-exercise performance supplementation on cardiovascular risk, blood lipids, and fasting blood glucose in resistance trained men

**DOI:** 10.1186/1550-2783-8-S1-P19

**Published:** 2011-11-07

**Authors:** Jeong-Su Kim, William K Mandler, David Thomas, Colin J Riley, Amber W Kinsey, Lynn B Panton, Michael J Ormsbee

**Affiliations:** 1 Department of Nutrition, Food, and Exercise Sciences. The Florida State University, Tallahassee, FL 32306, USA

## Background

Nutritional supplements intended for consumption in proximity to resistance exercise are extremely popular among young males and athletes. Common components of these products include caffeine, creatine monohydrate, β-Alanine (BA), citrulline, L-arginine, branched chain amino acids (BCAA), and whey protein in proprietary blends. To date, there have been no investigations of the potential health risks or benefits associated with consumption of these products over the course of a resistance training (RT) regimen despite anecdotal reports to health complications. The purpose of this study was to investigate the effect of the commercial sports nutritional supplements NO-Shotgun® (SHOT) and NO-Synthesize® (SYN) (Vital Pharmaceuticals, Inc., Davie, FL) on cardiovascular risk, blood lipids, and glucose in resistance trained men following 6weeks of supplementation and concurrent resistance exercise.

## Methods

Eight resistance trained men completed 6 weeks (3d/week)of periodized resistance training (RT) including one day eachfor arms/shoulders, legs/core, and chest/back. The participants were assigned to 1 of 2 groups (based on maximal voluntary contraction of the quadriceps (Biodex) to lean mass ratio). Group 1 (n=5; Performance Supplement; PS) consumedone serving of SHOT before and 1 serving of SYN immediately after each RT session and on non-RT days. Group 2 (n=3; Placebo; PL) consumedan isocaloric maltodextrin placebo (PL) before and immediately after each RT session and on non-RT days. Measurements included pre- and post-RT resting heart rate (HR) and blood pressure (SBP and DBP), fasting blood lipoproteinprofile and glucose (Cholestech LDX Analyzer; Cholestech Corp, Hayword, CA).Statistical analysis was conducted using a 2 x 2 repeated measures analysis of variance. Significance is set at p<0.05 and values reported as mean ± SE.

## Results

There were no significant time or group by time effects for HR, SBP, DBP either PS group or the PL group. Serum triglycerides (TRG) and glucose (GLU) did not differ between groups and remained unchanged following RT. Total cholesterol (TC) was higher (p=0.0027) pre- and post-RT for the PL group (PRE: PS, 134.2 ± 8.3 vs. PL,182.7± 3.4 mg/dl; POST: PS, 138.7 ± 19.0 vs. PL, 188.0±1.7 mg/dl), however, there was no time effect for either group. Low density lipoprotein (LDL) was higher (p=0.022) in the PL group pre- and post-RT (PRE: PS, 72.8 ± 12.6 vs. PL, 122.7± 11.3 mg/dl; POST: PS, 82.0 ± 9.7 vs. PL, 129.6± 6.7 mg/dl) but there was no time effect for either group. High density lipoprotein (HDL) was not different between groups before RT while there was a trend of group x time interaction (p=0.073) due to different directional responses in the PS(+10.3%)and PL group (-7.6 %) after RT.

## Conclusion

The consumption of SHOT and SYN performance supplements over the course of a 6-week RT regimen does not alter any of the measured cardiovascular health parameters, and may positively influence HDL levels. However, more participants are needed to improve statistical power and support these results.

